# Antipsychotic prescribing patterns on admission to and at discharge from a tertiary care program for treatment-resistant psychosis

**DOI:** 10.1371/journal.pone.0199758

**Published:** 2018-08-10

**Authors:** Lik Hang N. Lee, Ric M. Procyshyn, Randall F. White, Todd S. Woodward, William G. Honer, Alasdair M. Barr

**Affiliations:** 1 Department of Pharmacology, University of British Columbia, Vancouver, British Columbia, Canada; 2 Department of Psychiatry, University of British Columbia, Vancouver, British Columbia, Canada; Max-Planck-Institut fur Psychiatrie, GERMANY

## Abstract

Retrospective data were collected from 330 individuals who were treated at a tertiary care program for treatment-resistant psychosis between 1994 and 2010. The main objectives were to compare the use of antipsychotic monotherapy to polypharmacy and to characterize within-individual changes in treatment and symptomatology between admission and discharge. At admission, individuals who were prescribed only one antipsychotic were comparable to those who were prescribed at least two antipsychotics with regard to demographics and symptom severity. The use of psychotropic medications other than antipsychotics was also similar between the two groups. However, the magnitude of antipsychotic utilization was greater in individuals who were receiving antipsychotic polypharmacy. In addition, a greater proportion received excessive doses at admission. Similar findings were observed when the two antipsychotic prescribing practices were compared at discharge. Three important patterns were identified when investigating within-individual changes. First, fewer individuals were prescribed more than one antipsychotic at discharge. This was accompanied by a general decrease in the magnitude of antipsychotic utilization. Second, the number of individuals who were prescribed clozapine had increased by discharge. Most who were already prescribed clozapine at admission had their doses increased. Third, improvements in symptomatology were observed across all of the subscales included in the Positive and Negative Symptom Scale (PANSS); 57.9% of individuals experienced a relative reduction in total PANSS scores exceeding 20%. Based on these findings, it is possible to alleviate symptom severity while reducing antipsychotic utilization when patients are treated at a tertiary care program for treatment-resistant psychosis.

## Introduction

Since the introduction of chlorpromazine, antipsychotics have remained the primary form of pharmacotherapy for the treatment of psychosis. Yet even with the number of antipsychotics available, symptomatic remission is often not achieved despite the appropriate use of these medications. A significant proportion of individuals with psychosis will fail to attain an adequate clinical response. Such patients may be referred to as having refractory or treatment-resistant psychosis (TRP) [1] (although see [2]). Consensus diagnostic criteria for TRP have historically remained elusive [3], although there is an implicit agreement among researchers and clinicians that treatment resistance requires the continued persistence of symptoms despite at least two antipsychotic trials of adequate dose and duration [4]. While a lack of response to two antipsychotics does not necessarily preclude response to all other antipsychotics, an iterative process of trial and error may not be the best approach once treatment resistance, however defined, is established [3, 4]. Instead, referral to tertiary services may be the more appropriate course of action for many individuals with TRP.

Tertiary services differ from primary and secondary psychiatric care in that the staff practicing in these referral programs typically have greater clinical experience with TRP [5]. While clinical experience is best used as a supplement to evidence-based practice, few, if any, pharmacological strategies have proven to be more effective than the use of appropriately dosed antipsychotic monotherapy. For example, clozapine is the only antipsychotic to have consistently demonstrated clinical efficacy in TRP, with improvements in negative symptoms over the short term (i.e., less than 3 months) and improvements in positive symptoms over both the short and the long term [6]. While the putative pharmacological mechanism of action of clozapine is the same as all other antipsychotics—i.e., occupancy of dopamine D_2_ receptors in the mesolimbic pathway as an antagonist (or, in the case of aripiprazole, a partial agonist)—additional pharmacological properties [7] may account for the greater improvements in response rates and clinical outcomes that make it the only antipsychotic indicated for TRP. Unfortunately, clozapine remains underutilized in clinical practice, in part because of a lack of familiarity with the drug [8] and in part because of concerns about its hazardous adverse effects [9]. In some cases, pharmacological strategies that are lacking in evidence (e.g., antipsychotic polypharmacy) may be tried before clozapine is even attempted [10, 11].

Owing to the dearth of evidence in the literature for strategies other than clozapine monotherapy, it is important to determine which, if any, approaches may be effective in TRP when implemented in an inpatient tertiary care setting by an experienced clinical team. A previous pair of studies in the United Kingdom reported that the improvements in symptom severity following hospitalization at a tertiary care facility for TRP [12] coincided with changes in psychotropic medications [5]. Thus, it may be possible to make within-individual comparisons of treatment effectiveness by characterizing the strategies used at admission and discharge as we have demonstrated previously in youth [13].

In the present study, we analyzed the use of antipsychotics and other psychotropic medications among patients who were referred to a tertiary care program for TRP at Riverview Hospital in Coquitlam, British Columbia, Canada. Riverview served as the tertiary care psychiatric hospital for the entire province of British Columbia until 2012. The Refractory Psychosis Program (hereafter referred to as the “Program”) was established in 1988 to meet the demand for psychiatric beds dedicated to individuals with TRP who did not respond adequately to treatment in the community or at the referring hospital. Patients who were admitted to the Program underwent a comprehensive evaluation to confirm their diagnosis and assess the extent of their treatment resistance. Personalized treatment plans were then developed by a team of psychiatrists, psychologists, social workers, and psychiatric nurses. Using retrospective data from this Program, we compared the use of antipsychotics and other psychotropic medications at admission and discharge. In addition to these within-individual comparisons, we also compared those who were prescribed only one antipsychotic (i.e., antipsychotic monotherapy) and those who were prescribed at least two antipsychotics (i.e., antipsychotic polypharmacy) at both time points. Although there is little evidence to support the concurrent use of two or more antipsychotics, it remains a prevalent practice and is often associated with excessive antipsychotic utilization and an increased risk for adverse effects [14–17].

It was hypothesized that antipsychotic utilization, objectively measured using the Defined Daily Dose method (described in detail below), would be lower among individuals who were on antipsychotic monotherapy because dose-dependent adverse effects should deter excessive dosing. While it is certainly possible for individuals to receive lower doses of each antipsychotic when the drugs are prescribed as part of polypharmacy, the use of two or more antipsychotics typically results in excessive dosing when the cumulative antipsychotic dose is taken into consideration [18]. The majority of individuals were also hypothesized to have switched from antipsychotic polypharmacy to clozapine monotherapy over the course of hospitalization because clinicians at the Program would have greater familiarity with the drug and be better able to monitor patients for its rare but potentially life-threatening adverse effects. Such a finding would be consistent with a previous comparison of antipsychotic prescribing practices between a research ward (i.e., the Program) and general treatment wards at Riverview Hospital [19]; the relative frequency of antipsychotic polypharmacy was lower among individuals discharged from the research ward than from wards bearing a closer resemblance to referring hospitals. The speculated decrease in the number of antipsychotics prescribed at discharge should be accompanied by a reduction in the prevalence of excessive dosing for the same reason detailed above.

## Methods and materials

The clinical ethics committee at the University of British Columbia approved the study protocol which complies with the ethical standards laid down in the 1964 Declaration of Helsinki.

### Data collection

Retrospective data were abstracted from the charts of individuals who were admitted to the Program at Riverview Hospital between September 1994 and November 2010. The IRB waived the requirement for informed consent. Patients were between the ages of 17 and 64, did not have a primary diagnosis of substance abuse, and displayed persistent psychotic symptoms despite previous treatment with antipsychotics. Most were characterized as poorly responsive or refractory according to the May scale [20]. Individuals were only included in the study if the severity of their psychotic symptoms had been rated by clinicians around the time of admission and discharge using the Positive and Negative Syndrome Scale (PANSS) [21]. For most individuals, these ratings were made approximately 1–2 weeks following admission, prior to the consensus diagnostic conference, and around the time when these individuals were deemed ready for discharge. The PANSS is administered as a semi-formalized interview and consists of 30 items; seven to assess positive symptoms (i.e., delusions; conceptual disorganization; hallucinatory behavior; excitement; grandiosity; suspiciousness; hostility), seven to assess negative symptoms (i.e., blunted affect; emotional withdrawal; poor rapport; passive-apathetic social withdrawal; difficulty in abstract thinking; lack of spontaneity and flow of conversation; stereotyped thinking), and sixteen to assess general psychopathology (i.e., somatic concern; anxiety; guilt feelings; tension; mannerisms and posturing; depression; motor retardation; uncooperativeness; unusual thought content; disorientation; poor attention; lack of judgment and insight; disturbance of volition; poor impulse control; preoccupation; active social avoidance). The severity of each item is scored on a seven-point integer scale, with 1 being absent and 7 being extreme. As these ratings are made based on information from the preceding week, they are subject to change over time, making the PANSS an appropriate instrument for assessing the clinical outcome in those treated at the Program. Raters were trained routinely to ensure a high inter-rater reliability as described previously [22].

In addition to these ratings of symptom severity, psychiatric diagnosis (according to DSM-III-R or DSM-IV criteria), demographic data (i.e., diagnosis; sex; age at admission; prior clozapine trial), regularly scheduled medications at admission and discharge, and measures of functional outcome as assessed using both the social and occupational functioning assessment scale (SOFAS) and the global assessment of functioning (GAF) scale were recorded where available. *Pro re nata* (PRN) or ‘as needed’ medications were not included in the analyses. Individuals were excluded if they had a psychiatric diagnosis other than schizophrenia or schizoaffective disorder. If the individual had more than one admission to the Program, then data from their last available admission were used.

Data pre-processing was completed using R version 3.4.3 [23]. In addition to the pre-installed packages, those included in *tidyverse* (v. 1.2.1) [https://CRAN.r-project.org/package=tidyverse/] were used to prepare the data for analyses. Particular attention was paid to errors that could arise during data entry. For example, missing data, nonsensical data, and data arousing suspicion (e.g., antipsychotic doses that are not typically used in clinical practice) were identified in the electronic data and checked against physical records where possible.

### Antipsychotic utilization

The Defined Daily Dose (DDD) method developed by the World Health Organization (WHO) was used to quantify antipsychotic utilization at admission to and discharge from the Program. For each individual, the prescribed daily dose (PDD) of each antipsychotic was divided by its DDD, which represents “the assumed average maintenance dose per day for a drug used for its main indication in adults” [24]. For long-acting injectable antipsychotics, the PDD was determined by dividing the administered dose by the number of days between injections. If the individuals were prescribed more than one antipsychotic at a given time, the different quotients (or PDD:DDD ratios) were added together to quantify total antipsychotic utilization. If the total utilization is equal to 1, then the prescribed antipsychotic dose or doses are assumed to be appropriate for the treatment of schizophrenia. However, consistent with the criterion used in previous studies [14, 15, 25, 26], PDD:DDD ratios exceeding 1.5 were considered excessive.

### Statistical analyses

Statistical analyses were also conducted using R. A complete-case approach was used to handle missing data. All hypothesis testing was two-tailed. P-values less than 0.05 were considered to be statistically significant. All figures were generated using *ggplot2* (v. 2.2.1) [27], *cowplot* (v. 0.9.2) [28], and extrafont (v. 0.17) [29].

To compare antipsychotic prescribing practices (i.e., monotherapy vs. polypharmacy), the *coin* (v. 1.2–2) implementation of the Wilcoxon-Mann-Whitney test [30] was used to identify differences in the distributions of age, antipsychotic utilization, and symptom severity. The *exact2x2* (v. 1.5.2) implementation of Boschloo’s test [31] was used to identify associations between prescribing practices and sex, diagnosis, past use of clozapine meeting the criteria for a successful trial, and current use of psychotropic medications.

To characterize within-individual changes following treatment at the Program, the *coin* implementation of the Wilcoxon signed-rank test was used to compare antipsychotic utilization and symptom severity at admission and discharge. A mid-p variant of the McNemar test [32] was used to examine marginal homogeneity in 2x2 contingency tables involving either the use of clozapine or the antipsychotic prescribing practice employed at admission and discharge.

### Exploratory analyses

Within-individual and between-group comparisons of social, occupational, and psychological functioning were also made using scores from both the SOFAS and the GAF scale. A higher score on each of these 100-point scales indicates better overall functioning. The duration of treatment, best approximated by the number of days between PANSS assessments, was also compared between the antipsychotic monotherapy and polypharmacy groups at discharge. This measure was used in place of the total duration of hospitalization because the latter can be influenced by factors that are unrelated to clinical response. For example, discharge from the Program can be delayed if there are difficulties coordinating the continuation of care.

Results pertaining to within-individual changes in antipsychotic utilization were explored in greater detail. In addition to the use of the Wilcoxon signed-rank tests on subgroups of interest, the *coin* implementation of the Kruskal-Wallis test was used to compare groups of individuals who differed in terms of their patterns in treatment. The *Pairwise Multiple Comparison of Mean Ranks* (v.4.2) implementation of Dunn’s test (with Bonferroni adjustment) [33] was used for post-hoc analyses where necessary.

Lastly, logistic regression was used to identify potential predictors of antipsychotic polypharmacy and clozapine use at discharge. With the exception of the ‘excessive dosing’ variable, which was determined entirely by the magnitude of antipsychotic utilization, all bases of comparison between antipsychotic monotherapy and polypharmacy at admission (e.g., age; diagnosis; antipsychotic utilization; PANSS scores) were used as candidate variables for the two models (see comparisons in Table 1). Continuous variables were centered at their means before their inclusion in the models. Given the low number of events per variable for both outcomes, the least absolute shrinkage and selection operator (lasso) was used to reduce overfitting by shrinking the values of regression coefficients and setting some to zero, effectively accomplishing feature selection in the process [34]. This entire procedure, including the use of tenfold cross-validation to identify an appropriate tuning parameter, was completed using the *glmnet* package (v. 2.0–13) [35]. Since the variables that are included in each model can change depending on factors such as the value of the tuning parameter, those that are present in one model are not necessarily more important than those that have been excluded. Therefore, 1000 nonparametric bootstrap samples were generated for each of the two outcomes to determine the frequency with which each candidate variable was left out of the resulting models [34].

**Table 1:** Comparison of antipsychotic monotherapy and polypharmacy at admission.

Admission (n = 327)					
	Monotherapy	% of Group	Polypharmacy	% of Group	P-value
	n = 156 (47.7%)		n = 171 (52.3%)		
**Baseline Characteristics**					
Male	92	59.0%	103	60.2%	0.816
Female	64	41.0%	68	39.8%			
Age, median (Q1, Q3)	37 (25, 44)	-	36 (29, 44.5)	-	0.375		
Schizophrenia	105	67.3%	122	71.3%	0.450		
Schizoaffective Disorder	51	32.7%	49	28.7%			
**Antipsychotics**					
Prior Clozapine Trial	78	50.0%	111	64.9%	0.006
Typicals	48	30.8%	125	73.1%	< 0.001
Atypicals	108	69.2%	148	86.6%	< 0.001
Clozapine	26	16.7%	41	24.0%	0.121
Olanzapine	27	17.3%	57	33.3%	< 0.001
Quetiapine	26	16.7%	48	28.1%	0.015
Risperidone^a^	28	17.9%	56	32.7%	0.002
Utilization, median (Q1, Q3)^b^	1.25 (0.80, 2.00)	-	2.38 (1.55, 3.20)	-	< 0.001
Excessive dosing^c^	57	36.8%	129	75.9%	< 0.001
**Other Drugs**					
Anticholinergics	26	16.7%	40	23.4%	0.155
Antidepressants	33	21.2%	37	21.6%	1.000
Benzodiazepines	55	35.3%	52	30.4%	0.388
Mood Stabilizers	53	34.0%	71	41.5%	0.161
**Symptom Severity (PANSS ratings)**					
Positive Scale, median (Q1, Q3)	25 (21, 29)	-	25 (22, 30)	-	0.140
Negative Scale, median (Q1, Q3)	26 (21, 29.25)	-	25 (21.5, 28)	-	0.416
General Psychopathology Scale, median (Q1, Q3)	47 (40, 53)	-	47 (42, 52)	-	0.728
Total, median (Q1, Q3)	97.50 (83.75, 110.25)	-	98 (87, 109)	-	0.678

PANSS, positive and negative syndrome scale

^a^ oral and intramuscular (depot) formulations of risperidone were combined

^b^ two individuals were excluded because data on antipsychotic doses were missing; utilization was calculated using the defined daily dose method

^c^ n = 155 in the monotherapy group; n = 170 in the polypharmacy group (see b); excessive dosing was defined as utilization greater than 1.5

## Results

Data were collected from 330 patients; 232 (70.3%) had a diagnosis of schizophrenia and 98 (29.7%) had a diagnosis of schizoaffective disorder. In contrast to a previous study with this population [36], subtypes were not included in these analyses.

### Comparison of antipsychotic monotherapy and polypharmacy at admission

Of the 327 individuals who were prescribed antipsychotics at admission, 156 (47.7%) were on antipsychotic monotherapy and 171 (52.3%) were on polypharmacy. The majority of individuals treated with two or more antipsychotics (102; 59.6%) were prescribed a combination of typical and atypical antipsychotics, but 46 (26.9%) were treated exclusively with atypical antipsychotics and 23 (13.5%) were treated exclusively with typical antipsychotics.

Sex, age at admission, diagnosis, use of psychotropic medications other than antipsychotics, and severity of psychotic symptoms were not significantly different between those who were prescribed only one antipsychotic and those who were prescribed multiple antipsychotics (Table 1). Functioning was also comparable between the two groups when assessed using scores on both the SOFAS (median score for both groups: 31; Z = -0.143; P = 0.886) and the GAF scale (median score for both groups: 25; Z = 0.110; P = 0.913). However, antipsychotic polypharmacy was associated with past use of clozapine (OR = 1.85, P = 0.006) and greater use of antipsychotics by class (typical antipsychotics, OR = 6.11, P < 0.001; atypical antipsychotics, OR = 2.86, P < 0.001) and by agent (i.e., olanzapine, OR = 2.39, P < 0.001; quetiapine, OR = 1.95, P = 0.015; risperidone, OR = 2.23, P = 0.002) at admission. Antipsychotic utilization (i.e. PDD:DDD ratio) was significantly different between the two groups (Z = -8.00, P < 0.001). The use of multiple antipsychotics was associated with excessive dosing (OR = 5.41; P < 0.001). Nonetheless, the use of clozapine was not different between the two groups (OR = 1.58, P = 0.121) despite the use of more antipsychotics in the polypharmacy group.

### Comparison of antipsychotic monotherapy and polypharmacy at discharge

Of the 328 patients who were prescribed antipsychotics at discharge, 273 (83.2%) were prescribed one antipsychotic and the remaining 55 (16.8%) were prescribed two antipsychotics. The median number of days between admission and discharge PANSS assessments was 155 (interquartile range: 96 to 247), but when divided into the two groups, the median duration was only 147 days (interquartile range: 93.0 to 220.5) in the antipsychotic monotherapy group compared to 246 days (interquartile range: 136.5 to 388.0) in the polypharmacy group (Z = -3.73; P < 0.001). Clozapine was the most frequently prescribed antipsychotic in both groups, but risperidone was also commonly prescribed to those using more than one antipsychotic. Most individuals in the polypharmacy group (42, 76.4%) were treated exclusively with atypical antipsychotics, with the combination of clozapine and risperidone being the most prevalent (49.1% of all combinations). Only 12 (21.8%) were treated with a combination of atypical and typical antipsychotics. One person (1.8%) was treated exclusively with typical antipsychotics.

Again, the only major differences observed between the two groups at discharge were restricted to their use of antipsychotics (Table 2). Antipsychotic polypharmacy was associated with a prior clozapine trial (OR = 2.20, P = 0.014) and greater use of antipsychotics by class (i.e., typical antipsychotics, OR = 2.25, P = 0.028; atypical antipsychotics, OR = 7.43, P = 0.023). However, when antipsychotics were no longer grouped according to their propensity to cause extrapyramidal symptoms, antipsychotic polypharmacy was found to be associated with the use of risperidone (OR = 19.66, P < 0.001), but not with the use of olanzapine (OR = 0.84, P = 1.000), quetiapine (OR = 1.85, P = 0.314), or clozapine (OR = 0.60, P = 0.156). Antipsychotic utilization was different between the two groups (Z = -7.21, P < 0.001); antipsychotic polypharmacy was once again significantly associated with excessive dosing (OR = 7.83, P < 0.001).

**Table 2:** Comparison of antipsychotic monotherapy and polypharmacy at discharge.

Discharge (n = 328)					
	Monotherapy	% of Group	Polypharmacy	% of Group	P-value
	n = 273 (83.2%)		n = 55 (16.8%)		
**Baseline Characteristics**					
Male	163	59.7%	32	58.2%	0.875	
Female	110	40.3%	23	41.8%		
Age, median (Q1, Q3)	36 (27, 45)	-	38 (30.0, 42.5)	-	0.555	
Schizophrenia	188	68.9%	40	72.7%	0.611	
Schizoaffective Disorder	85	31.1%	15	27.3%		
**Antipsychotics**					
Prior Clozapine Trial^a^	149	54.8%	40	72.7%	0.014	
Typicals	33	12.1%	13	23.6%	0.028	
Atypicals	240	87.9%	54	98.2%	0.023	
Clozapine	165	60.4%	39	70.9%	0.156	
Olanzapine	29	10.6%	5	9.1%	1.000	
Quetiapine	14	5.1%	5	9.1%	0.314	
Risperidone^b^	24	8.8%	36	65.5%	< 0.001	
Utilization, median (Q1, Q3)^c^	1.33 (1.00, 1.85)	-	2.27 (1.70, 2.78)	-	< 0.001	
Excessive dosing^d^	107	39.5%	46	83.6%	< 0.001	
**Other Drugs**					
Anticholinergics	15	5.5%	2	3.6%	0.729	
Antidepressants	46	16.9%	11	20.0%	0.556	
Benzodiazepines	25	9.2%	9	16.4%	0.136	
Mood Stabilizers	78	28.6%	15	27.3%	1.000	
**Symptom Severity (PANSS ratings)**					
Positive Scale, median (Q1, Q3)	18 (15, 21)	**-**	20 (16, 23)	**-**	0.062
Negative Scale, median (Q1, Q3)	22 (18, 25)	**-**	22 (19, 27)	**-**	0.540
General Psychopathology Scale, median (Q1, Q3)	40 (34, 46)	**-**	39 (36, 47)	**-**	0.730
Total, median (Q1, Q3)	80 (68, 91)	**-**	80 (71.5, 92.5)	**-**	0.407

PANSS, positive and negative syndrome scale

^a^ one individual from the monotherapy group was excluded because of missing data

^b^ oral and intramuscular (depot) formulations of risperidone were combined

^c^ two individuals were excluded because data on antipsychotic doses were missing; utilization was calculated using the defined daily dose method

^d^ n = 271 in the monotherapy group (see b); excessive dosing was defined as utilization greater than 1.5

While there is little to suggest that the two groups differ in terms of symptom severity, scores on the GAF scale appeared to be different at discharge. Functioning appears to be slightly worse among individuals who were prescribed two or more antipsychotics (median: 33; interquartile range: 28.00 to 37.75) than among those who were prescribed only one antipsychotic (median: 35; interquartile range: 30 to 40). This difference (Z = 2.46; P = 0.014) was not corroborated by a comparison of SOFAS scores around the time of discharge (median for both groups: 38; Z = 0.543; P = 0.587).

### Within-individual change in antipsychotic prescribing practice

**Fig 1:**
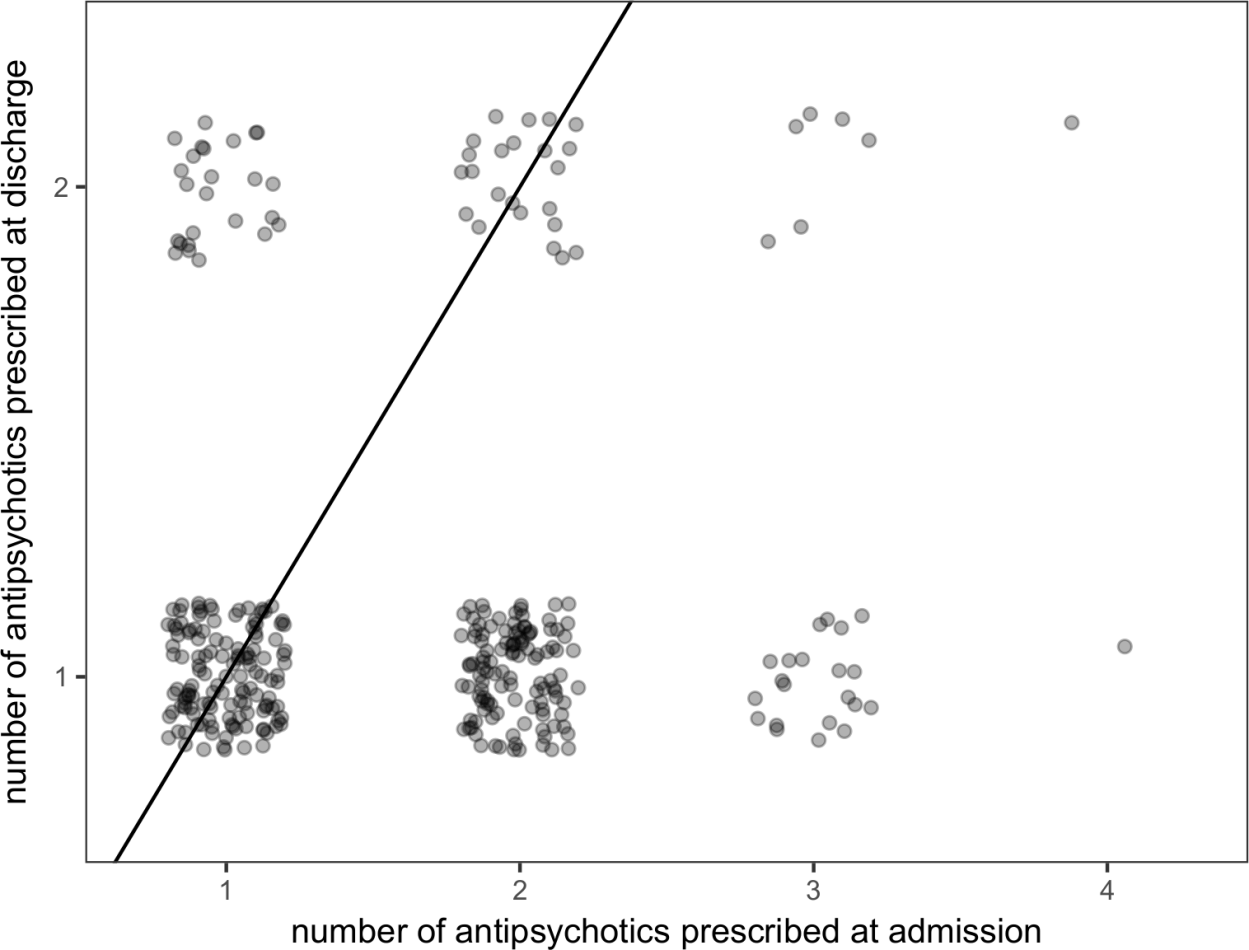
Number of antipsychotics prescribed at admission and discharge. Each individual who was prescribed at least one antipsychotic at admission and discharge (n = 325) is represented by a point. Points that are clustered around the line represent individuals who were prescribed the same number of antipsychotics at admission and discharge. Those found above / to the left of the line had more antipsychotics at discharge. Those found below / to the right of the line had fewer antipsychotics at discharge.

Most individuals who were prescribed at least two antipsychotics at admission were prescribed only one antipsychotic at discharge (Fig 1). Even among the 30 individuals who were on antipsychotic polypharmacy at both admission and discharge, seven saw a reduction in the number of antipsychotics prescribed. Although there were individuals that were switched from monotherapy to polypharmacy over the course of hospitalization, the largest possible increase in the number of antipsychotics prescribed was limited to one since no individuals were prescribed more than two antipsychotics at discharge. Of the 325 patients who were prescribed antipsychotics at admission and discharge, 139 (42.8%) were switched from polypharmacy at admission to monotherapy at discharge, while 25 (7.7%) were switched from monotherapy at admission to polypharmacy at discharge. The probability of switching from polypharmacy to monotherapy over the course of hospitalization was evidently different than that of switching from monotherapy to polypharmacy (χ^2^ = 79.24, df = 1, mid-p < 0.001).

### Within-individual change in antipsychotic utilization

**Fig 2:**
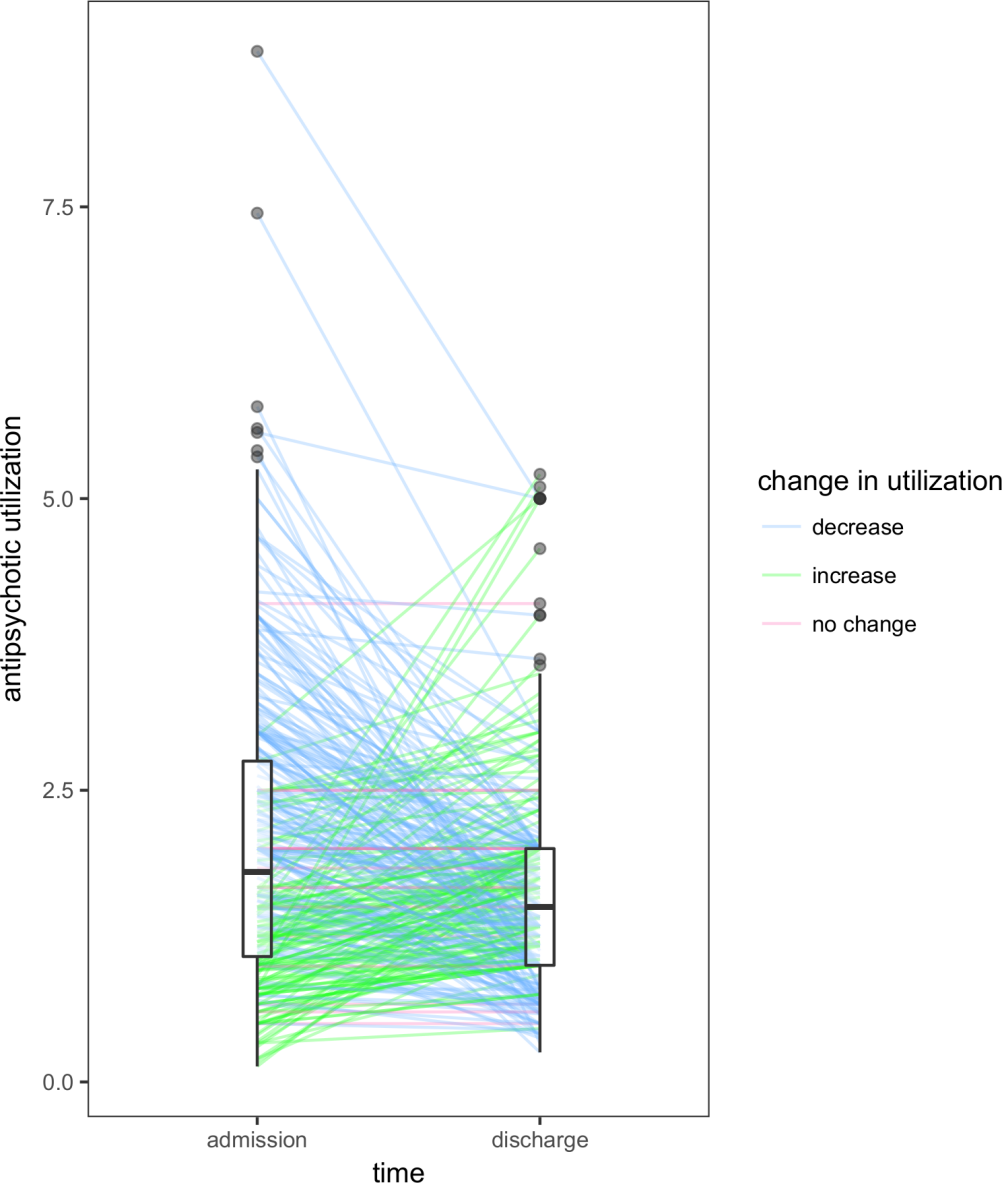
Within-individual change in antipsychotic utilization. Each individual who had data on antipsychotic utilization at admission and discharge (n = 321) is represented by a line. Boxplots depict distribution of antipsychotic utilization at their respective time points. Median utilization at admission was 1.8 (interquartile range: 1.075 to 2.750). Median utilization at discharge was 1.5 (interquartile range: 1.0 to 2.0). Utilization was determined using the defined daily dose method.

Of the 321 individuals who had data on antipsychotic dosing at admission and discharge, the median within-individual change was -0.2 (interquartile range, -1.167 to 0.367). Therefore, in addition to the decrease in the number of antipsychotics prescribed, a decrease in utilization was also observed in the majority of individuals (Fig 2).

**Fig 3:**
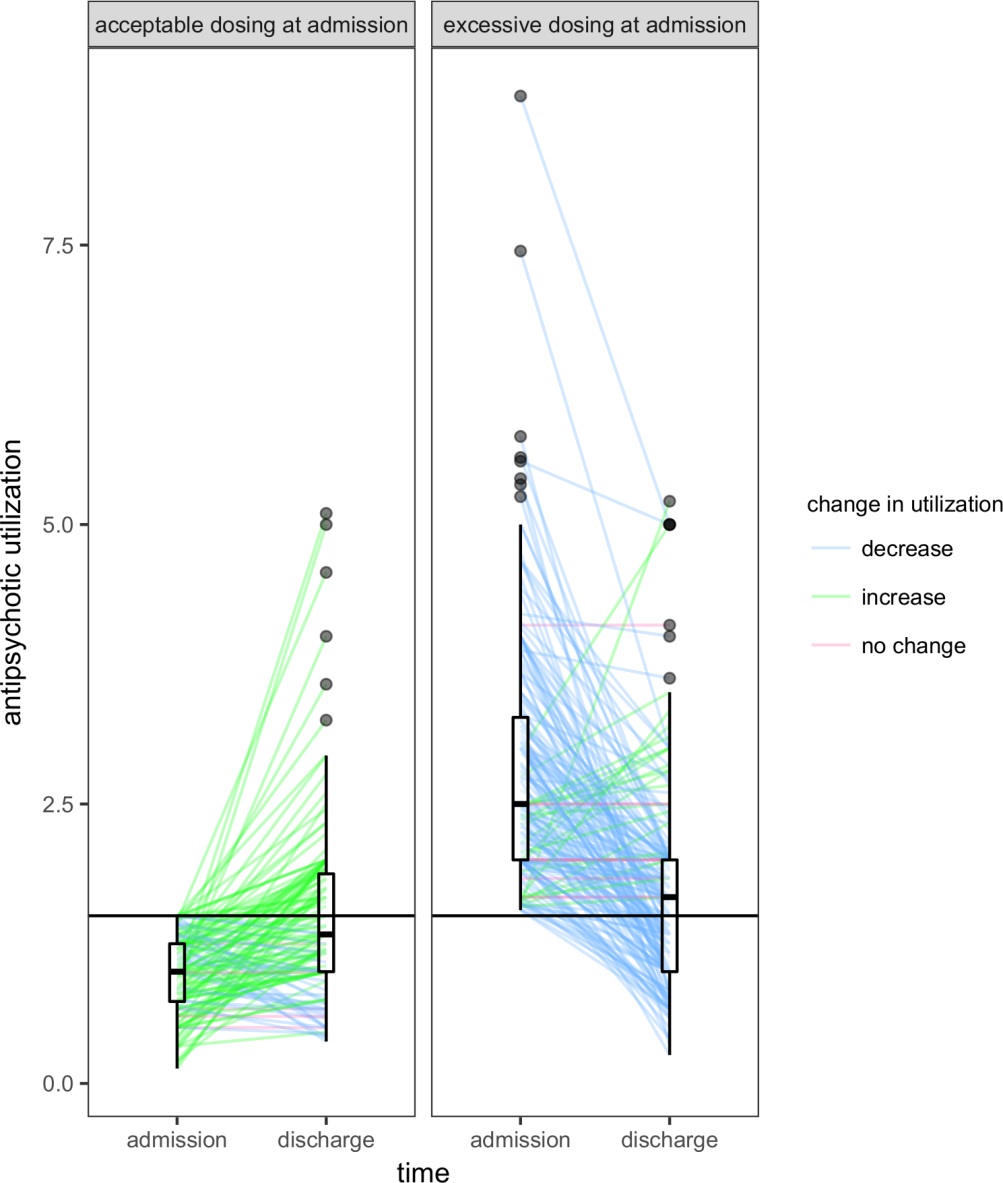
Within-individual change in antipsychotic utilization by extent of utilization at admission. Each individual who had data on antipsychotic utilization at admission and discharge (n = 321) is represented by a line. Boxplots depict distribution of antipsychotic utilization at their respective time points. For individuals with acceptable dosing at admission (n = 138), the median utilization was 1.0 (interquartile range: 0.73 to 1.25) at admission and 1.33 (interquartile range: 1.000 to 1.875) at discharge. For individuals with excessive dosing at admission (n = 183), the median utilization was 2.5 (interquartile range: 2.000 to 3.275) at admission and 1.67 (interquartile range: 1.0 to 2.0) at discharge. The horizontal line corresponding to an antipsychotic utilization of 1.5 represents the threshold for excessive antipsychotic utilization.

Further exploration revealed a difference in the direction of change when individuals were grouped according to the extent of utilization at admission (Fig 3). Of the 183 individuals (57.0%) whose antipsychotic utilization at admission was deemed excessive, the median within-individual difference was found to be -1.0 (interquartile range: -1.750 to -0.296). Of the 138 individuals (43.0%) whose utilization was deemed to be acceptable at admission, the median within-individual difference was found to be 0.333 (interquartile range: 0.000 to 0.829). In both cases, the distribution of these within-individual changes was not symmetric about 0 (P < 0.001). Consequently, the tendency toward a decrease in antipsychotic utilization across the entire cohort following treatment at the Program can be largely attributed to the contributions of those receiving excessive doses at admission.

**Fig 4:**
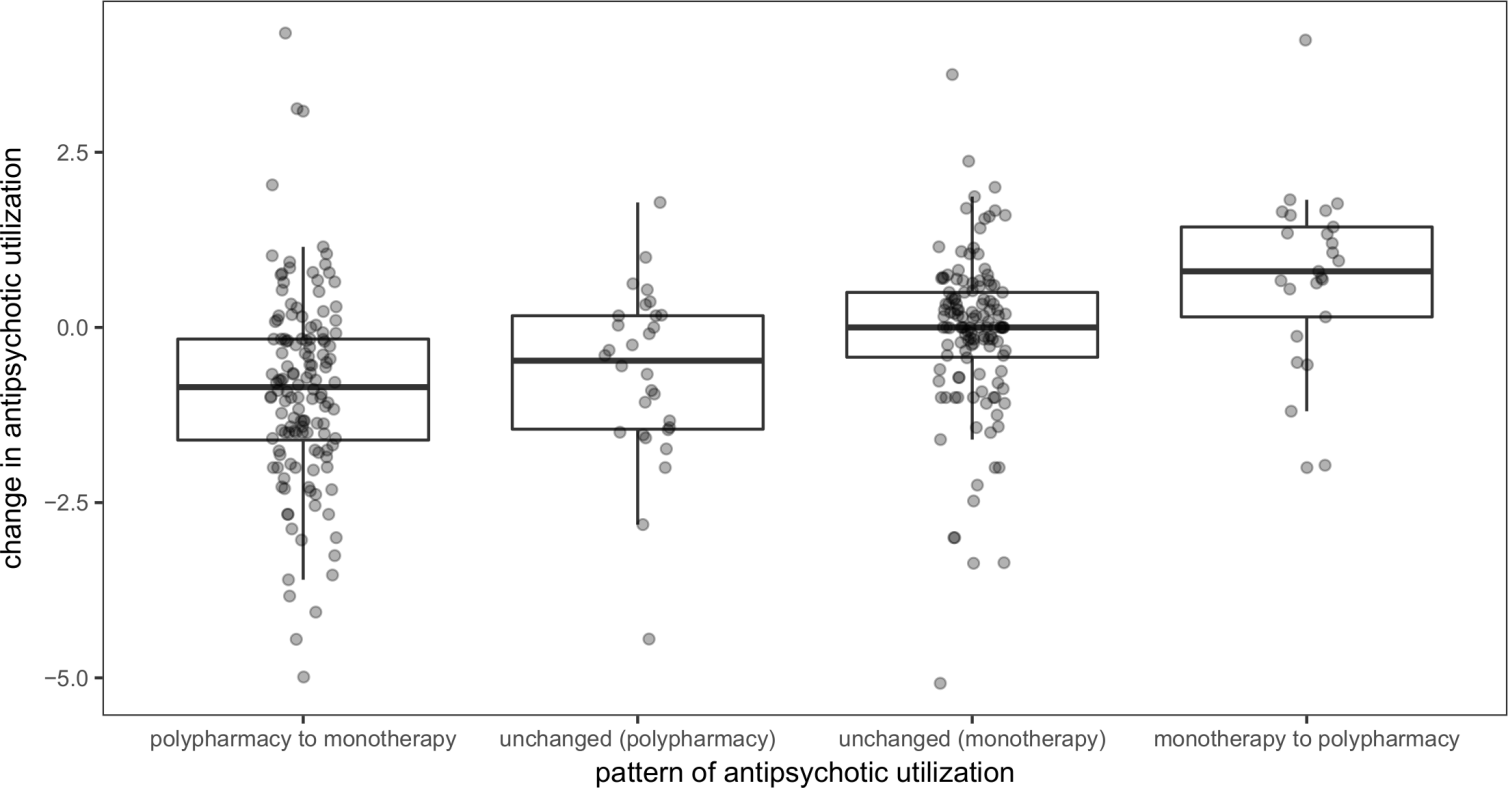
Comparison of within-individual change in antipsychotic utilization by antipsychotic prescribing pattern. Each individual who had data on antipsychotic utilization at admission and discharge (n = 321) is represented by a point and grouped according to the prescribing pattern. Boxplots depict the distribution of this change. For individuals switching from polypharmacy to monotherapy (n = 136), the median change in utilization was -0.85 (interquartile range: -1.61 to -0.17). For individuals who remained on polypharmacy (n = 30), the median change in utilization was -0.48 (interquartile range: -1.45 to 0.17). For individuals who remained on monotherapy (n = 130), the median change in utilization was 0.00 (interquartile range: -0.43 to 0.50). For individuals switching from monotherapy to polypharmacy (n = 25), the median change in utilization was 0.80 (interquartile range: 0.15 to 1.43). Utilization was determined using the defined daily dose method.

Taking this analysis further, when the individuals were grouped according to antipsychotic prescribing practices at admission and discharge, a significant difference emerged in terms of the change in antipsychotic utilization (χ^2^ = 54.13, df = 3, P < 0.001) (Fig 4). Individuals who were switched from antipsychotic monotherapy to polypharmacy were significantly different from those who switched from polypharmacy to monotherapy (P < 0.001) and those who remained on polypharmacy (P < 0.001) since most experienced an increase rather than a decrease in antipsychotic utilization. The only other pairwise comparison that resulted in a statistically significant difference was between those who switched from polypharmacy to monotherapy and those who remained on monotherapy (P < 0.001). Change in antipsychotic utilization did not vary significantly when comparing the different clozapine prescribing patterns.

### Within-individual change in clozapine treatment

Of the 325 individuals who were prescribed antipsychotics at admission and discharge, 10 (3.1%) were prescribed clozapine at admission but not at discharge and 145 (44.6%) were prescribed clozapine at discharge but not at admission (χ^2^ = 117.58, df = 1, mid-p < 0.001). The difference in magnitude suggests that the clinicians at the Program favored the use of clozapine in managing TRP.

**Fig 5:**
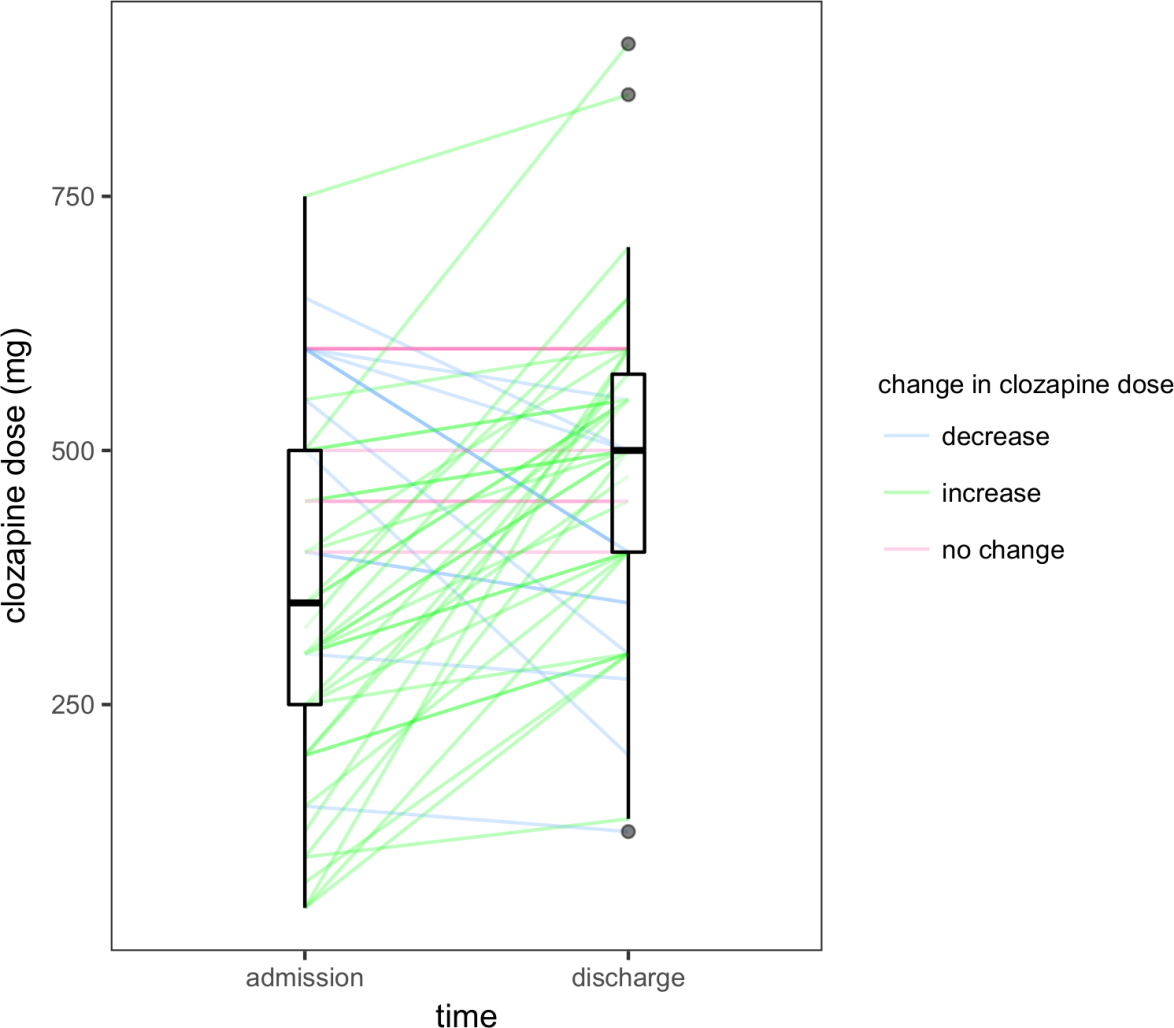
Within-individual change in clozapine utilization. Each individual who had data on clozapine utilization at admission and discharge (n = 56) is represented by a line. Boxplots depict distribution of clozapine doses at their respective time points. The median dose was 350 mg (interquartile range: 250 mg to 500 mg) at admission and 500 mg (interquartile range: 400 mg to 575 mg) at discharge. The defined daily dose of clozapine is 300 mg.

There were 57 individuals who were prescribed clozapine at admission and discharge. Data on the doses used at both time points were available for all but one of these individuals. The change in dose was unlikely to be symmetric about 0 (Z = -3.83, P < 0.001); the median within-individual difference was 100 mg (interquartile range, 0 to 231.25 mg; Fig 5). Thus, the optimization of clozapine therapy typically involved an up-titration of the daily dose.

### Within-individual change in symptom severity and functioning

**Fig 6:**
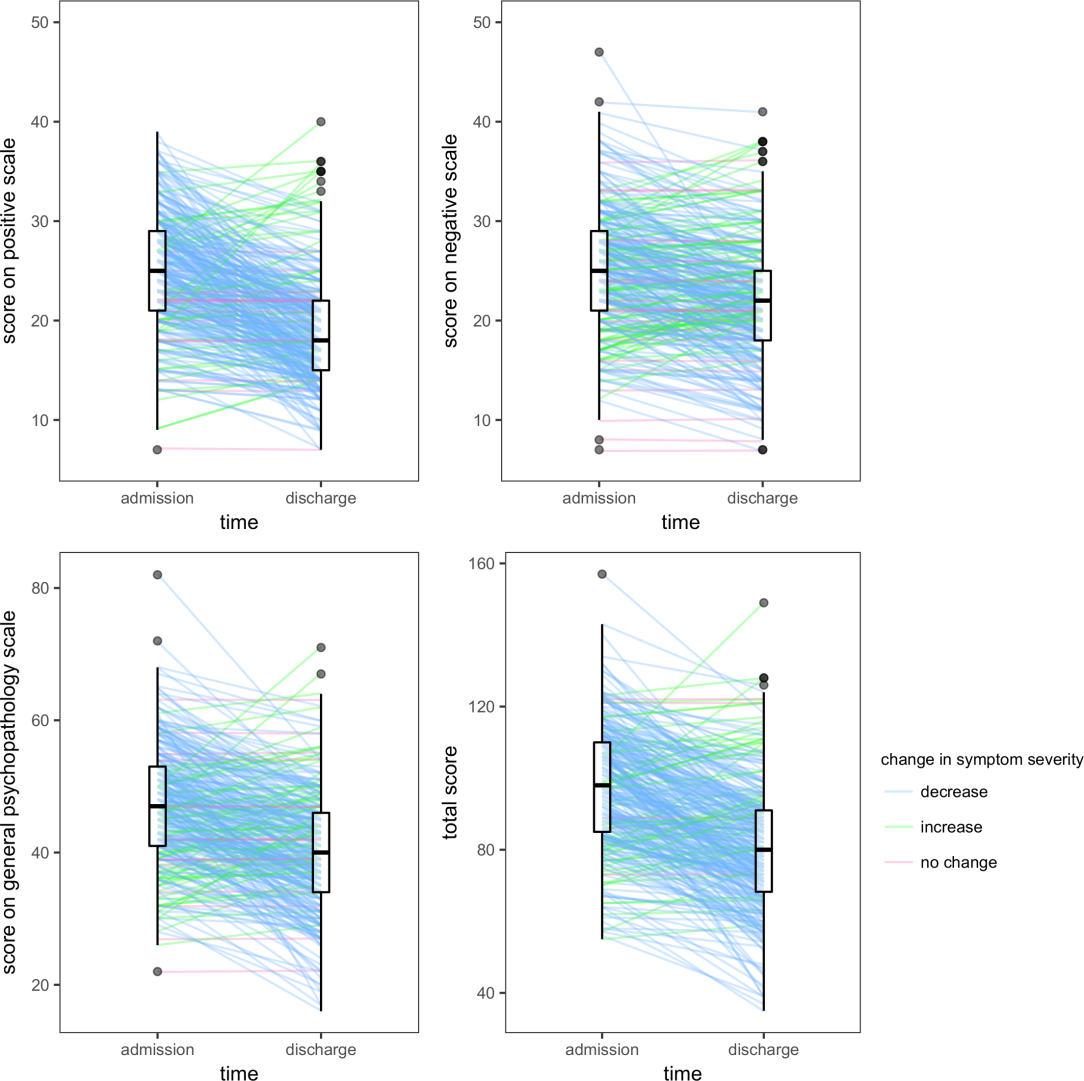
Within-individual change in psychotic symptom severity. Each individual who was assessed using the Positive and Negative Syndrome Scale (n = 330) is represented by a line. Higher scores reflect greater symptom severity. Boxplots depict distribution of ratings at their respective time points. The median rating on the positive scale was 25 (interquartile range: 21 to 29) at admission and 18 (interquartile range: 15 to 22) at discharge. The median rating on the negative scale was 25 (interquartile range: 21 to 29) at admission and 22 (interquartile range: 18 to 25) at discharge. The median rating on the general psychopathology scale was 47 (interquartile range: 41 to 53) at admission and 40 (interquartile range: 34 to 46) at discharge. The median rating across all scales was 98 (interquartile range: 85 to 110) at admission and 80 (interquartile range: 68 to 91) at discharge.

Median within-individual differences in symptom severity were -5 (interquartile range: -10 to -2) on the positive scale, -3 (interquartile range: -6 to 0) on the negative scale, -7 (interquartile range: -13 to -1) on the general psychopathology scale, and -16 (interquartile range: -27 to -6) across all scales. Since none of the distributions of these within-individual differences were likely to be symmetric about 0 (P < 0.001), it is clear that most individuals had lower ratings of symptom severity around the time of discharge (Fig 6). A relative reduction in total PANSS scores exceeding 20% was observed in 191 individuals (57.9%).

A similar pattern was observed for measures of overall functioning. The median within-individual difference on the SOFAS and the GAF scale were 7 (interquartile range: 3 to 13) and 9 (interquartile range: 4 to 15), respectively (both P < 0.001).

### Predictors of antipsychotic polypharmacy and clozapine use at discharge

**Fig 7:**
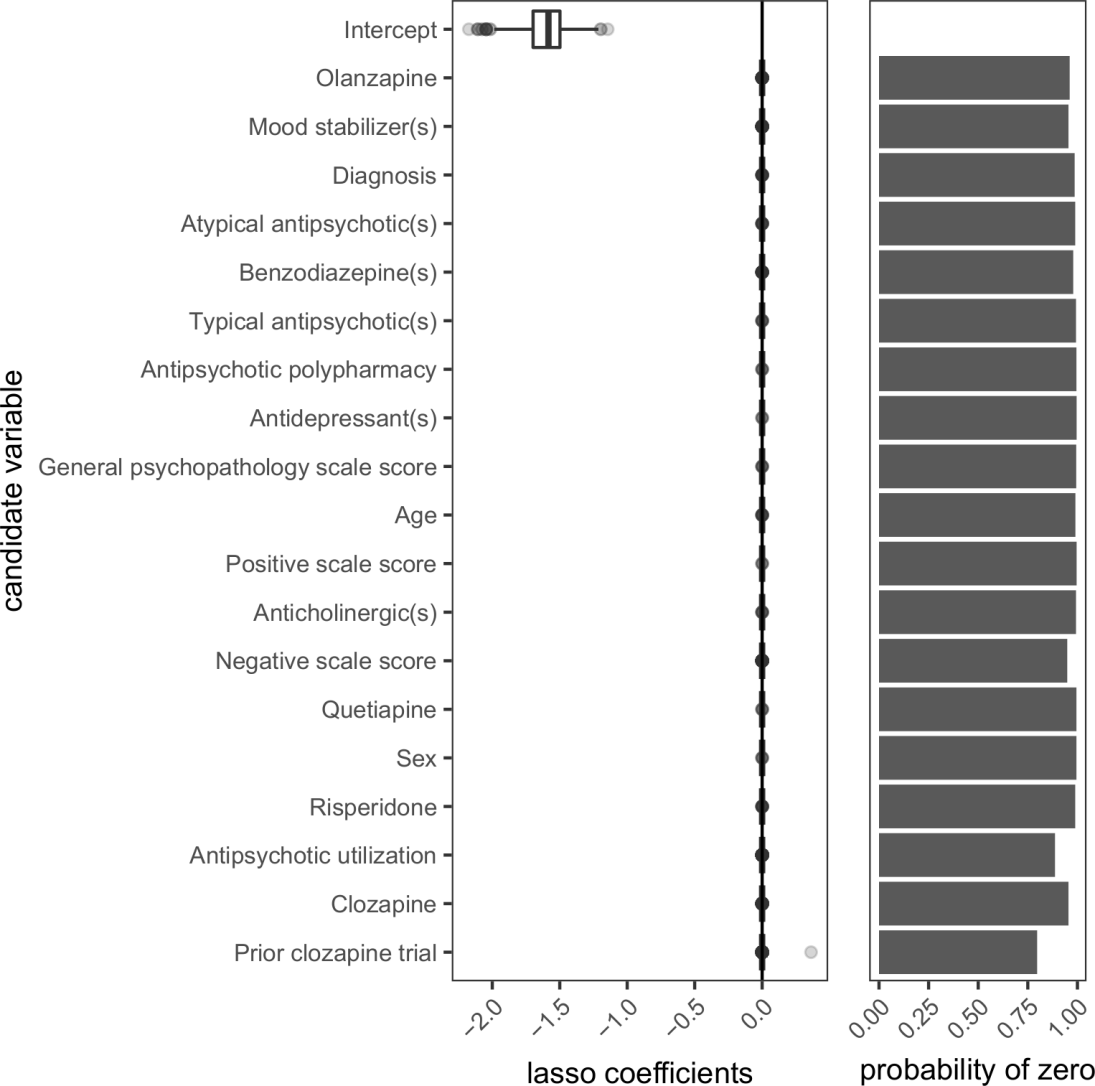
Distribution of regression coefficient estimates for predictors of antipsychotic polypharmacy at discharge. Lasso-regularized logistic regression was performed on 1000 bootstrap samples (each of size 321) to determine the values of the regression coefficient estimates and the probability with which each coefficient was set to zero. Candidate variables were those collected at admission and are presented from top to bottom in increasing order of the mean of the lasso coefficients. Age, antipsychotic utilization, positive scale score, negative scale score, and general psychopathology scale score are continuous variables. All other variables are binary. Diagnoses of schizophrenia were coded as 0 and schizoaffective disorder as 1. Males were coded as 0 and females as 1. Values of 0 for the remaining variables can be interpreted as ‘no’ or ‘absent’ whereas values of 1 can be interpreted as ‘yes’ or ‘present’.

Using the original data, none of the candidate variables entered the model that was intended for the prediction of antipsychotic polypharmacy at discharge. Even among the candidate variables that were occasionally included in the models trained on bootstrap data, estimates of the regression coefficients were almost always approximately 0 (Fig 7).

**Fig 8:**
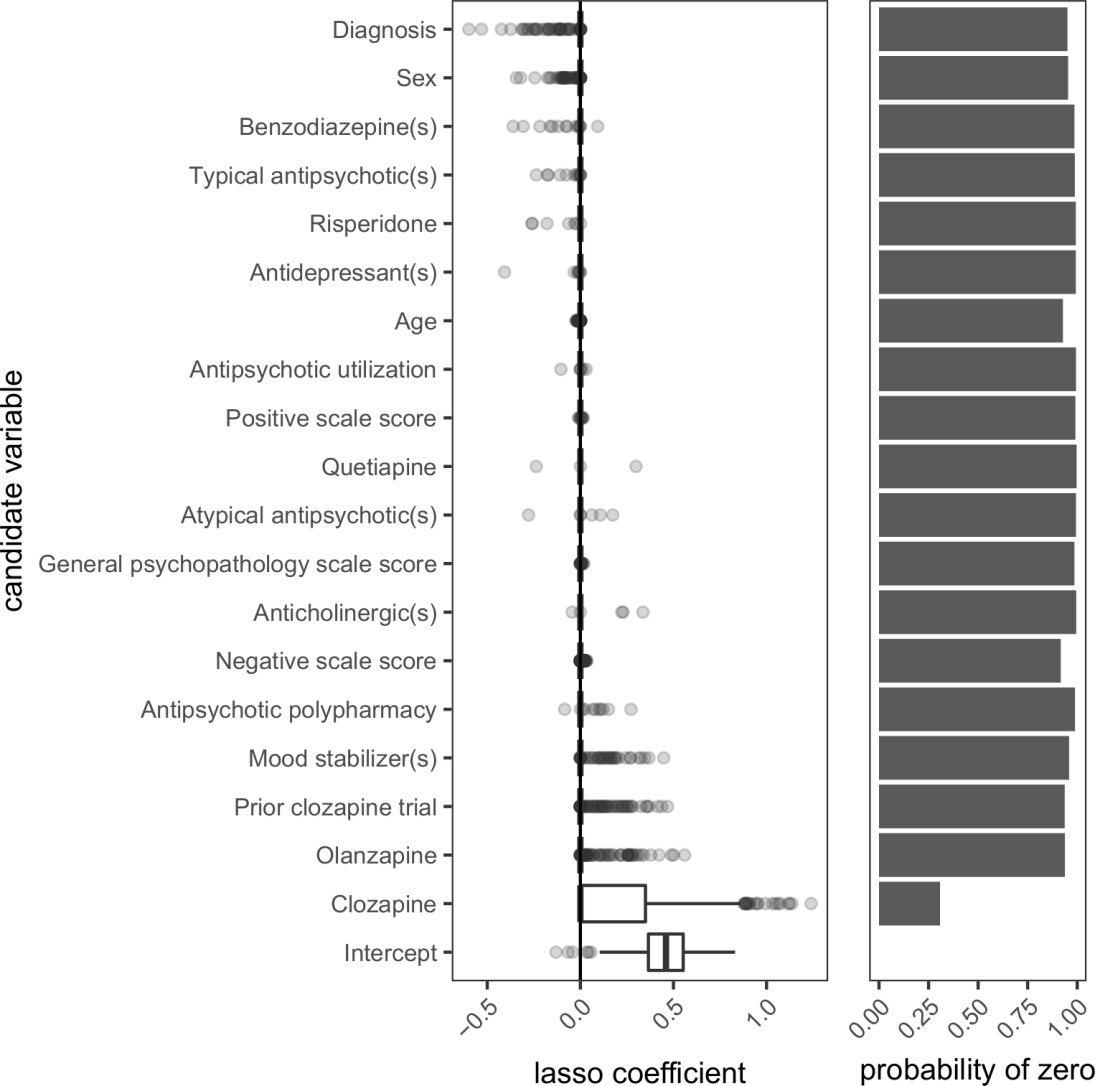
Distribution of regression coefficient estimates for predictors of clozapine use at discharge. Lasso-regularized logistic regression was performed on 1000 bootstrap samples (each of size 321) to determine the values of the regression coefficient estimates and the probability with which each coefficient was set to zero. Candidate variables were those collected at admission and are presented from top to bottom in increasing order of the mean of the lasso coefficients. Age, antipsychotic utilization, positive scale score, negative scale score, and general psychopathology scale score are continuous variables. All other variables are binary. Diagnoses of schizophrenia were coded as 0 and schizoaffective disorder as 1. Males were coded as 0 and females as 1. Values of 0 for the remaining variables can be interpreted as ‘no’ or ‘absent’ whereas values of 1 can be interpreted as ‘yes’ or ‘present’.

The only variable to enter the model predicting the use of clozapine at discharge was the use of clozapine at admission which had a regression coefficient estimate of 0.584. In 1000 bootstrap samples, this variable was excluded (i.e., set to zero) 308 times, resulting in a mean lasso coefficient estimate of 0.190 (Fig 8). In comparison, all other variables were excluded at least 917 times.

## Discussion

The primary goal of this retrospective study was to analyze the use of antipsychotics and other psychotropic medications in the 330 individuals who were treated at an inpatient tertiary care program for TRP. The findings indicate that the prevalence of antipsychotic polypharmacy decreased between admission and discharge. This occurred alongside a reduction in antipsychotic utilization. In parallel, clozapine use increased between admission and discharge, while symptom severity, measured using the PANSS, exhibited a significant reduction.

Aside from the expected differences related to the use of antipsychotics, individuals who were prescribed one antipsychotic were not found to differ from those who were prescribed multiple antipsychotics around the time of admission. This is in contrast to the findings in our previous study that was conducted in a community sample [18] where we observed an association between antipsychotic polypharmacy and the use of anticholinergics. However, since we did not record PRN medications in the present study, it is possible that the majority of anticholinergics were prescribed in this manner at the Program and so escaped our analysis. Another important finding was the lack of association between antipsychotic polypharmacy and the use of clozapine. Unlike other atypical antipsychotics such as olanzapine, quetiapine, and risperidone, the proportion of individuals who were prescribed clozapine at admission was similar between the two groups. This likely reflects a reluctance to prescribe clozapine as part of a treatment regimen involving two or more antipsychotics, which is consistent with the lack of support for clozapine-antipsychotic polypharmacy [36]. Be that as it may, the manner in which the other antipsychotics were used highlights the underutilization of clozapine in TRP. Prevalent use of antipsychotic polypharmacy in the community and in referring hospitals may have delayed the initiation of clozapine in individuals who had yet to try the antipsychotic [10].

At discharge, the only differences observed between the two groups were also limited to the use of antipsychotics. But unlike before, risperidone was the only atypical antipsychotic whose use was associated with antipsychotic polypharmacy. This finding likely reflects the interest in using risperidone as an adjunct to clozapine that existed around the time when many of these individuals were discharged from the Program. A major clinical trial involving participants at Riverview Hospital would later find this strategy to produce no additional clinical benefits in this population [37].

As noted above, two important patterns emerged in terms of pharmacological treatment at the Program. First, the majority of individuals who were prescribed multiple antipsychotics at admission experienced a reduction in the number of antipsychotics prescribed at discharge. Second, the extent of antipsychotic utilization was typically lower at discharge than it was at admission. This was most apparent among individuals who were admitted on an excessive dose or doses of antipsychotics and those who had switched from antipsychotic polypharmacy at admission to monotherapy at discharge. In contrast, most individuals who went from antipsychotic monotherapy to polypharmacy saw increases in antipsychotic utilization, which is consistent with the link between antipsychotic polypharmacy and excessive dosing [14, 38] In a related finding, the majority of individuals who had acceptable doses at admission saw increases in antipsychotic utilization by discharge. In fact, 56 of 138 transitioned from acceptable to excessive antipsychotic utilization. This suggests that the utilization needed for a clinical response may be greater in TRP than it is for treatment-responsive psychosis [39]. Consequently, the term “excessive” dosing should be used carefully when applied to TRP. In fact, the standard of care employs the use of serum levels rather than the current PDD as a means of guiding dose adjustments.

For the majority of patients who were not prescribed clozapine at admission, the drug was initiated during hospitalization and presumably titrated to effect by discharge. For the majority of individuals who were prescribed clozapine at both time points, the dose at discharge was greater than that at admission. Most importantly, more than half of the patients admitted to the Program were prescribed clozapine by discharge, thus ameliorating the issue of clozapine underutilization in primary and secondary care [40]. This pattern of clozapine utilization is comparable to another naturalistic, retrospective study [5]. Among 153 patients with treatment-resistant schizophrenia who were admitted to a tertiary care program in the United Kingdom, 90 were prescribed clozapine at discharge compared to 47 at admission. Moreover, 41 of the 47 individuals who were prescribed clozapine at admission continued to be treated with clozapine at discharge. This program also achieved a reduction in polypharmacy, but when clozapine was co-prescribed with another antipsychotic, the second drug was usually amisulpride (unavailable in Canada) or quetiapine rather than risperidone.

A decrease in psychotic symptom severity was observed across all domains over the course of hospitalization. Based on an equipercentile linking procedure between scores on the PANSS and scores on the CGI severity scale [41], the median PANSS score was closest to a rating of “markedly ill” at admission and a rating of “moderately ill” at discharge. Similarly, based on the linking of the absolute change in total PANSS score to the CGI improvement scale [42], the median change in total PANSS scores most closely resembled someone whose psychotic symptoms were “minimally improved”. To put this into perspective, this degree of improvement is not typically considered a response in most randomized controlled trials [4]. However, the rate of response may be lower than expected because the individuals referred to the Program tend to be those with the greatest degree of treatment resistance in the entire province. As the options in the pharmacological management of TRP are limited, it is understandable that a response is not attained in all individuals admitted to the Program. As mentioned, 57.9% of the individuals who were included in this study experienced a relative reduction in total PANSS scores that exceeded 20%. This is the more commonly used threshold for establishing response in TRP [4].

Based on the lasso-regularized logistic regression models, there does not appear to be a single variable available at admission that can be used as an adequate predictor of antipsychotic polypharmacy at discharge. However, clozapine use at admission may have some value in predicting its use at discharge. Unfortunately, since the present study was a retrospective chart review and only the data available in charts and supporting documentation were available, it is impossible to conclude with certainty whether all individuals underwent an adequate clozapine trial at some point following their admission to the Program, particularly if they were prescribed other antipsychotics at admission. Given the nature of the Program, it is reasonable to assume that the drug would have been offered to those who have not had an adequate trial in the past, but for those who have had an unsuccessful trial prior to their admission, there are no guarantees that a re-challenge would have been attempted. Certain reasons for treatment discontinuation may merit a re-challenge under close surveillance [43], but ultimately, these decisions are best made on a case-by-case basis. Future studies would benefit from documenting the use of clozapine during hospitalization and the reason for its discontinuation (if applicable). This would enable researchers to further characterize individuals based on their response to clozapine and to determine if this potential predictor is of any practical relevance. If clozapine was less likely to be prescribed to those who were not already taking the drug at admission, then it would make sense for its use at admission to predict future use at discharge. However, this finding would not be particularly illuminating because the reason for its inclusion in the model may have more to do with patterns in antipsychotic prescribing than the potential for clinical response. On the other hand, if clozapine were prescribed to all individuals, the greater likelihood of being discharged on clozapine if admitted on clozapine would likely reflect characteristics specific to the two subgroups: most who were admitted on clozapine likely tolerated the drug but were in need of optimization; but, some who were not prescribed clozapine at admission likely had to stop the treatment owing to adverse effects associated with its use [44–50].

There are a number of additional limitations in the present study. While it may be tempting to characterize the noted changes in antipsychotic treatment as definitive pathways to symptomatic improvement, this would be a gross oversimplification of the treatment received at the Program. Many forms of non-pharmacological therapy that were not captured in our study likely contributed to the improvements in symptom severity and overall functioning. Moreover, the data is only a crude approximation of the course of treatment since they were extracted from two time points that are believed to best capture suboptimal (i.e., that at admission) and optimized treatment (i.e., that at discharge). Some patients were started on multiple antipsychotics and discharged on a single antipsychotic agent. This could be due to treatment rationalization or clozapine prescription at discharge, but the exact reason remains undetermined as such information was not available in the chart notes. We cannot conclude, with certainty, whether the use of only one antipsychotic at discharge was the result of treatment rationalization or the use of clozapine. But while this may appear to be an unsatisfactory response, we believe that 1) transitioning from antipsychotic polypharmacy to monotherapy and 2) switching to clozapine both represent an evidence-based approach to the treatment of refractory psychosis. Consequently, it may be difficult to parse the difference between the two. If someone were to be discharged from the Program with clozapine being the only antipsychotic, then it is reasonable to assume that this was the treatment option that produced the best outcome out of all those that were tried during their hospitalization. We do not believe that clozapine would be prescribed as the only antipsychotic at discharge for the sake of prescribing clozapine.

The external validity of these results may be limited since the data were collected from a single site. Recall that a previous study had found the prescribing patterns at the Program to be different from those of the general treatment wards at the same hospital [19]. However, given the requirements for a referral to the Program (e.g., treatment resistance) and the prevalent use of clozapine at the time of discharge, it may be a worthwhile endeavor to replicate this study using data from other wards or clinics specializing in the use of clozapine.

One final point to consider is the method used to standardize antipsychotic doses for comparison. Patel et al. (2013)[51] have grouped all available methods for comparing antipsychotic doses into two major categories: calculated methods and consensus methods. As the names suggest, those belonging to the first category were developed using clinical data whereas those belonging to the second category are based entirely on expert opinion.

In the absence of a gold standard, the DDD method was used to compare antipsychotic utilization in this study. As described above, the DDD is “the assumed average maintenance dose per day for a drug used for its main indication in adults” [24]. These values are assigned by the WHO International Working Group for Drug Statistics Methodology after consulting relevant information. The dose ranges and dosing information found in product monographs (e.g., summary of product characteristics; highlights of prescribing information) are examples of the type of information used to inform the decision-making process. Although the DDD for a given drug can be amended at any time, changes are rarely made unless they are judged to be absolutely necessary because the stability of these values enable long-term studies on drug utilization.

The main advantage to using the DDD method is the ability to compare the utilization of most, if not all, antipsychotic [52]. For example, pericyazine and prochlorperazine were among the drugs prescribed at admission, but the values required for comparing their doses may not have been established for some consensus methods given the decline in their use. Conversely, there are comparisons involving newer antipsychotics that cannot be made using some calculated methods [53] because of a lack of appropriate data from which to estimate these values.

However, despite being classified as a calculated method, the lack of transparency in how the Working Group arrives at each value can engender perceptions of arbitrariness [51]. Furthermore, unlike the alternatives, the DDD method is not meant for establishing therapeutic equivalence [24]. That is, while two individuals with the same PDD:DDD ratio can be thought of as having been prescribed an equal amount of antipsychotics, the relative efficacy of these two treatments may differ. However, measures of antipsychotic utilization that have been determined using the DDD method have been found to be significantly correlated with measures of dose equivalence when applied to patient data[54, 55]. But Leucht et al. (2016) [53] are correct in stating that further study is required before the notion of interchangeability among methods is entertained.

Nonetheless, the patterns here suggest that optimization of antipsychotic treatment at a program where the clinical team has extensive experience with treatment resistance typically involves both the initiation and optimization of clozapine treatment and a departure from antipsychotic polypharmacy and excessive utilization. These procedures are largely consistent with evidence-based medicine from randomized clinical trials in TRP and appear to generalize to at least one other tertiary care program. Future prospective studies of antipsychotic use at tertiary care programs will be helpful in understanding how individualized treatment plans are determined.
